# What do haematological cancer survivors want help with? A cross-sectional investigation of unmet supportive care needs

**DOI:** 10.1186/s13104-015-1188-7

**Published:** 2015-06-06

**Authors:** Alix E Hall, Rob W Sanson-Fisher, Marita C Lynagh, Flora Tzelepis, Catherine D’Este

**Affiliations:** Priority Research Centre for Health Behaviour, Faculty of Health, Wing 4 HMRI Building, The University of Newcastle and Hunter Medical Research Institute, University Drive, Callaghan, NSW 2308 Australia; Priority Research Centre for Health Behaviour, Faculty of Health, The University of Newcastle and Hunter Medical Research Institute, University Drive, Callaghan, NSW 2308 Australia; National Centre for Epidemiology and Population Health (NCEPH), Research School of Population Health, ANU College of Medicine, Biology and Environment, The Australian National University, Canberra, ACT 0200 Australia

**Keywords:** Haematological cancer survivors, Unmet needs, Supportive care needs, Psychosocial, Oncology

## Abstract

**Background:**

This study aimed to identify the most prevalent unmet needs of haematological cancer survivors.

**Methods:**

Haematological cancer survivors aged 18–80 years at time of recruitment were selected from four Australian state cancer registries. Survivors completed the Survivor Unmet Needs Survey. The most frequently reported “high/very high” unmet needs items were identified, as well as characteristics associated with the three most prevalent “high/very high” unmet needs reported by haematological cancer survivors.

**Results:**

A total of 715 eligible survivors returned a completed survey. “Dealing with feeling tired” (17%), was the most frequently endorsed “high/very high” unmet need. Seven out of the ten most frequently endorsed unmet needs related to emotional health. Higher levels of psychological distress (e.g., anxiety, depression and stress) and indicators of financial burden as a result of cancer (e.g., having used up savings and trouble meeting day-to-day expenses due to cancer) were consistently identified as characteristics associated with the three most prevalent “high/very high” unmet needs.

**Conclusions:**

A minority of haematological cancer survivors endorsed a “high/very high” unmet need on individual items. Additional emotional support may be needed by a minority of survivors. Survivors reporting high levels of psychological distress or those who experience increased financial burden as a result of their cancer diagnosis may be at risk of experiencing the most prevalent “high/very high” unmet needs identified by this study.

## Background

Haematological cancers are a diverse group of cancers. Some are acute and fast growing, while others are chronic in nature [1]. Treatment types are variable, ranging from intense, inpatient treatments, such as stem cell or bone marrow transplant, to regimes of “watchful-waiting” [1]. Survival rates for some haematological cancers have improved in a number of countries [2–4]. As people are living longer after a cancer diagnosis it is vital that cancer care reduces the impact of cancer experienced by survivors [5, 6]. A cancer survivor has been defined as a person diagnosed with cancer from the time of diagnosis until death [7].

Supportive care is an integral component of healthcare [8, 9] and involves the provision of necessary services to meet patients’ needs [8]. This includes physical, emotional, social, psychological, informational, spiritual and practical needs [8]. Designing programs and services that best address the needs of cancer survivors has been identified as a priority [6]. Information from cancer survivors themselves is crucial for developing and delivering appropriate supportive care services to cancer survivors.

Few studies have explored the supportive care needs of haematological cancer survivors [10]. Such research has been limited by small sample sizes, a lack of population-based samples and a failure to use comprehensive and standardized needs assessment tools [10]. The authors recently assessed the top unmet needs items of Australian and Canadian population-based samples of haematological cancer survivors using a comprehensive needs assessment tool [11]. However, these findings focused on survivors from one Australian state only [11]. To increase the generalizability of the findings a more comprehensive assessment of haematological cancer survivors’ unmet needs, to include several Australian states, is needed.

Understanding what areas haematological cancer survivors require help with will facilitate improvements to the quality and appropriateness of supportive care provided to this population. Furthermore, identifying the sub-groups of survivors who may be at risk of experiencing a higher level of unmet need will help to deliver and target timely supportive care to those who need it most. Extending on our previous research, this brief report aimed to identify the ten most frequently endorsed “high/very high” unmet needs among a large sample of haematological cancer survivors, as well as the characteristics associated with the three most prevalent “high/very high” unmet needs reported by haematological cancer survivors.

## Methods

### Study design

Adult haematological cancer survivors were recruited from four Australian state-based cancer registries and asked to complete a self-report pen-and-paper survey.

### Participants

Eligible survivors were aged 18–80 years at time of recruitment and diagnosed with a haematological cancer.

### Procedure

The standard recruitment procedures of each cancer registry were used. Registry A used a direct patient contact model of recruitment, whereby all eligible survivors were identified by the registry and mailed a questionnaire package. Non-responders were mailed a reminder questionnaire package approximately 4 weeks later (methods reported elsewhere) [12]. The three remaining registries (B, C & D) employed a rolling recruitment method, where survivors were identified and approached on an ongoing basis between September 2011 and July 2012. Passive clinician consent was sought from the treating clinicians of all eligible survivors. This model of recruitment involved the registries notifying each eligible survivor’s treating clinician of intent to contact the survivor regarding the study. If clinicians did not object to the registry contacting their patient in a specified period of time, the registry contacted the survivor, informed them of the study [13], and requested their consent to have their contact details released to the researchers. Survivors who consented to having their contact details released from the registry to the researchers were sent a questionnaire package by the researchers. Non-responders were mailed a second questionnaire package approximately 4 weeks later and contacted by telephone after a further 4 weeks. Return of a completed survey was taken as informed consent to participate. Ethics approval was obtained from the University of Newcastle Human Research Ethics Committee and the relevant ethics committees associated with each cancer registry.

### Measures

The Survivors Unmet Needs Survey (SUNS) contains a total of 89 items and assesses unmet needs across five domains: Financial Concerns (11 items), Emotional Health (33 items), Access and Continuity of Care (22 items), Information (8 items) and Relationships (15 items) [14]. Each item is scored from 0 (‘no unmet need’) to 4 (‘very high unmet need’) [14]. The SUNS has been found to be a relevant instrument for use with haematological cancer survivors with good psychometric properties in this population [15].

Age, sex, cancer type, postcode, diagnosis date and other disease and demographic information were obtained directly from the cancer registries for those survivors who provided consent. Other socio-demographic information was obtained from the self-report survey. De-identified non-participant data relating to age at diagnosis, cancer type, rural/urban location at diagnosis and sex were also obtained from the cancer registries.

### Statistical analysis

Chi-squared analyses were used to compare characteristics of participants and non-participants. For each of the 89 items of the SUNS ‘high’ and ‘very high’ responses were combined. The number and percentage of survivors reporting a “high/very high” unmet need for each of the 89 items was calculated with 95% confidence intervals and ranked from highest to lowest [11, 14]. The ten most highly endorsed “high/very high” unmet needs were identified. Separate logistic regression analyses were used to identify demographic, disease, treatment, social and psychological characteristics associated with each of the three most prevalent “high/very high” unmet needs items reported by haematological cancer survivors. Variables with a p-value of 0.2 or less on χ^2^ analyses were included in the logistic regression analysis and a backwards stepwise method was used to remove variables with a p-value of 0.1 or more on the likelihood ratio test. The Hosmer–Lemeshow goodness of fit test was used to assess the fit of each model.

## Results

### Participants

Of the 1957 eligible survivors approached by the registries 715 (37%) returned completed surveys. A flowchart detailing the recruitment of the 715 haematological cancer survivors from each of the four cancer registries for the larger study is published elsewhere [16]. The sociodemographic and disease characteristics of participants, including: time since diagnosis, sex, cancer type and age-group at diagnosis, are shown in Table 1. Age at diagnosis and cancer type were statistically significantly different between participants and non-participants.

**Table 1:** Participant demographic characteristics

Characteristic^a^	N (%) (total sample = 715)
Median time since diagnosis	35 months (SD = 18.5).
Sex	
Male	390 (59%)
Female	275 (41%)
Cancer type	
Non-Hodgkin’s lymphoma	387 (58%)
Leukaemia	130 (20%)
Myeloma	107 (16%)
Hodgkin’s lymphoma	41 (6.2%)
Age group at diagnosis (years)	
15–39	53 (8.0%)
40–49	71 (11%)
50–59	174 (26%)
60–69	235 (35%)
70–80	132 (20%)

^a^Totals may not add to equal the final sample size due to missing values.

### Top unmet needs

The ten most frequently endorsed “high/very high” unmet needs items are presented in Table 2. Seven of the top ten most frequently endorsed “high/very high” unmet needs were from the Emotional Health domain, two from the Relationships domain and one from the Financial Concerns domain.

**Table 2:** Top ten ‘high/very high’ unmet needs reported by haematological cancer survivors

Rank	Unmet need	N (%) (n = 715)	SUNS domain
1	Dealing with feeling tired	116 (17%)	Emotional Health
2	Coping with having a bad memory or lack of focus	99 (14%)	Emotional Health
3	Dealing with feeling worried (anxious)	94 (13%)	Emotional Health
4	Dealing with changes in my physical ability	93 (13%)	Emotional Health
4	Finding someone to talk to who understands and has been through a similar experience	93 (13%)	Relationships
6	Dealing with people who expect me to be “back to normal”	87 (12%)	Relationships
7	Dealing with feeling stressed	86 (12%)	Emotional Health
7	Dealing with not feeling able to set future goals or make long-term plans	86 (12%)	Emotional Health
9	Finding car parking that I can afford at the hospital or clinic	83 (12%)	Financial Concerns
9	Dealing with being told I had cancer	83 (12%)	Emotional Health

NB: observations with missing data ranged from 2.1 to 5.3% across the 89 unmet needs items.

As shown in Table 2 “Dealing with feeling tired” was the most frequently reported unmet need. Survivors reporting that they had used up their savings as a result of cancer and treatment reported higher odds [OR 3.0; 95% CI 1.73, 5.14; likelihood ratio (LR) χ^2^ = 15.1, df = 1, *p* < 0.001] of experiencing a “high/very high” level of unmet need in regards to dealing with feeling tired compared to those survivors who did not report such financial burden. Survivors who reported above normal levels of depression (OR 3.9; 95% CI 2.16, 7.17; LR χ^2^ = 19.79, df = 1, *p* < 0.001), or anxiety (OR 2.7; 95% CI 1.50, 4.99; LR χ^2^ = 10.53, df = 1, *p* = 0.001), or stress (OR 3.6; 95% CI 1.95, 6.78; LR χ^2^ = 16.48, df = 1, *p* < 0.001) had significantly higher odds of experiencing a “high/very high” level of unmet need in relation to this item.

“Coping with having a bad memory or lack of focus” was the second most prevalent “high/very high” unmet need (Table 2). Survivors aged 50–59 years at diagnosis reported higher odds of experiencing a “high/very high” unmet need regarding this item (OR 4.0; 95% CI 1.40, 11.60; LR χ^2^ = 14.63, df = 4, *p* = 0.01) than those aged 70 years and over at diagnosis. Those with a high school or lower education reported significantly higher odds (OR 3.4; 95% CI 1.47, 7.97; LR χ^2^ = 8.87, df = 2, *p* = 0.01) than survivors with a university degree or higher qualification. Survivors who reported using up their savings as a result of cancer and treatment had higher odds of reporting a high level of need for this item compared to those survivors who did not (OR 2.3; 95% CI 1.07, 5.02; LR χ^2^ = 4.45, df = 1, *p* = 0.04). Survivors reporting above normal levels of depression (OR 3.7; 95% CI 1.87, 7.40; LR χ^2^ = 13.64, df = 1, *p* < 0.001) or stress (OR 10.1; 95% CI 5.11, 20.0; LR χ^2^ = 47.56, df = 1, *p* < 0.001) had significantly higher odds of experiencing a high level of unmet need in relation to coping with having a bad memory or lack of focus compared to survivors with normal levels of depression or normal levels of stress, respectively. Although not statistically significant at the 5% level, survivors in a partnered relationship had marginally higher odds (OR 2.2; 95% CI 0.99, 4.97; LR χ^2^ = 4.03, df = 1, *p* = 0.04) of reporting a “high/very high” unmet need for this item than those survivors who were single.

The third most highly endorsed unmet need was “dealing with feeling worried (anxious)” (Table 2). Survivors reporting having trouble meeting day to day expenses due to their cancer and treatment had higher odds (OR 3.1; 95% CI 1.47, 6.47; LR χ^2^ = 8.67, df = 1, *p* = 0.003) of reporting a “high/very high” unmet need in relation to this item than those who did not. Survivors who reported higher than normal levels of depression (OR 4.3; 95% CI 2.16, 8.62; LR χ^2^ = 17.04, df = 1, *p* < 0.001) or stress (OR 8.7; 95% CI 4.48, 16.90; LR χ^2^ = 43.63, df = 1, *p* < 0.001) had higher odds of reporting a “high/very high” level of unmet need on this item compared to their counterparts.

## Discussion

Relatively few haematological cancer survivors endorsed a high or very high unmet need on individual items from the SUNS. This finding is consistent with our investigation of Australian and Canadian haematological cancer survivors [11] as well as prior research assessing the unmet needs of haematological [17] and heterogeneous samples [14, 18] of cancer survivors. This data suggests that the care provided to haematological cancer survivors may be adequate in meeting the majority of their needs.

Health care providers should aim to provide haematological cancer survivors with supportive care that specifically addresses their unmet needs. In this study “dealing with feeling tired” was the most frequently endorsed “high/very high” unmet need. Previous research has identified cancer-related fatigue as a prevalent symptom experienced by haematological cancer survivors [19–21]. In line with clinical practice guidelines health care providers should assess all haematological cancer survivors for cancer-related fatigue and provide appropriate support, as recommended by clinical practice guidelines, to those experiencing such symptoms [22].

The majority of the top ten most frequently endorsed “high/very high” unmet needs were from the emotional health domain. This finding is consistent with previous research assessing the unmet needs of cancer survivors, with most of the top unmet needs identified in these studies belonging to emotional or psychological domains [11, 18]. Methodologically rigorous intervention trials aimed at identifying strategies that successfully reduce the most prevalent “high/very high” unmet needs of haematological cancer survivors may be warranted. However, a recent review found that few interventions have been effective in reducing the unmet needs of cancer patients [23]; although most of the previous research has not been conducted with haematological cancer survivors, further highlighting the need for research in this area.

This study found that elevated levels of psychological symptoms (either depression, anxiety or stress) and increased financial burden as a result of having cancer, (e.g. having used up their savings and trouble meeting day-to-day expenses), were characteristics consistently associated with haematological cancer survivors’ three most frequently reported “high/very high” unmet needs. This is similar to previous research conducted by the authors, which identified elevated psychological symptoms and increased financial burden as a result of cancer to be associated with haematological cancer survivors reporting a “high/very high” level of unmet need on seven or more items of the SUNS [16]. These results suggest that such sub-groups of haematological cancer survivors maybe at increased risk of experiencing “high/very high” unmet needs and additional support should be provided to these specific sub-groups. In addition, support targeted towards the most prevalent unmet needs identified by haematological cancer survivors may be most beneficial in alleviating unmet needs particularly among the most vulnerable sub-groups. Health care providers should consider using a psychometrically valid and reliable, as well as relevant measure, such as the SUNS, to assist them in identifying the specific areas haematological cancer survivors wish to receive additional support with. Such information should be used to direct and provide appropriate support.

Several limitations of this study should be acknowledged. The current sample was not representative of the entire population of haematological cancer survivors, with non-participants and participants differing with regard to age at diagnosis and cancer type. It is important to be cautious when generalising from these results to the wider population of haematological cancer survivors. While the response rate of 37% was low, this is comparable to several other psychosocial surveys utilising population-based cancer registries [14, 18]. In addition, extensive efforts were made to increase the response rates in the current study, including the undertaking of a randomised controlled trial to increase response rates from one of the state cancer registries [12] and conducting follow-up reminders with non-responders.

Despite these limitations this study provides important and much needed information on the supportive care needs of haematological cancer survivors. With a sample of over 700 survivors this is the largest study we are aware of that assesses the specific supportive care unmet needs of haematological cancer survivors [10]. This study also includes a large heterogonous, population-based sample of adult haematological cancer survivors, providing data from most sub-groups of haematological cancer survivors. Finally, the use of a comprehensive and relevant needs assessment tool [15] helps to ensure that the data provide an accurate reflection of the most prevalent unmet needs of haematological cancer survivors. Such information should be used by health care providers and researchers to improve the supportive care provided to haematological cancer survivors.

## Conclusion

Only a minority of haematological cancer survivors endorsed a “high/very high” unmet need on individual items. This finding may indicate that current supportive care is sufficient for meeting the majority of needs for most haematological cancer survivors. However, additional support, particularly in the area of emotional support may be needed by a minority of survivors. In particular, haematological cancer survivors who report high levels of psychological distress or experience increased financial burden as a result of their cancer diagnosis may be sub-groups of survivors at risk of experiencing an increased number of “high/very high” unmet needs items.

## Abbreviations

SUNS: Survivor Unmet Needs Survey
SD: standard deviation
